# Increased inflammation, oxidative stress and mitochondrial respiration in brown adipose tissue from obese mice

**DOI:** 10.1038/s41598-017-16463-6

**Published:** 2017-11-22

**Authors:** Martín Alcalá, María Calderon-Dominguez, Eduviges Bustos, Pilar Ramos, Núria Casals, Dolors Serra, Marta Viana, Laura Herrero

**Affiliations:** 1 Facultad de Farmacia, Universidad CEU San Pablo, Madrid, Spain; 2 Department of Biochemistry and Physiology, School of Pharmacy and Food Sciences, Institut de Biomedicina de la Universitat de Barcelona (IBUB), Universitat de Barcelona, E-08028 Barcelona, Spain; 3 Centro de Investigación Biomédica en Red de Fisiopatología de la Obesidad y la Nutrición (CIBEROBN), Instituto de Salud Carlos III, E-28029 Madrid, Spain; 4 Basic Sciences Department, Faculty of Medicine and Health Sciences, Universitat Internacional de Catalunya (UIC), E-08195 Sant Cugat del Vallés, Barcelona, Spain; 5 Present Address: Service of Development of Medicines (SDM), School of Pharmacy and Food Sciences, Universitat de Barcelona, E-08028 Barcelona, Spain

## Abstract

Obesity is associated with severe metabolic diseases such as type 2 diabetes, insulin resistance, cardiovascular disease and some forms of cancer. The pathophysiology of obesity-induced metabolic diseases has been strongly related to white adipose tissue (WAT) dysfunction through several mechanisms such as fibrosis, apoptosis, inflammation, ER and oxidative stress. However, little is known of whether these processes are also present in brown adipose tissue (BAT) during obesity, and the potential consequences on mitochondrial activity. Here we characterized the BAT of obese and hyperglycemic mice treated with a high-fat diet (HFD) for 20 weeks. The hypertrophic BAT from obese mice showed no signs of fibrosis nor apoptosis, but higher levels of inflammation, ER stress, ROS generation and antioxidant enzyme activity than the lean counterparts. The response was attenuated compared with obesity-induced WAT derangements, which suggests that BAT is more resistant to the obesity-induced insult. In fact, mitochondrial respiration in BAT from obese mice was enhanced, with a 2-fold increase in basal oxygen consumption, through the upregulation of complex III of the electron transport chain and UCP1. Altogether, our results show that obesity is accompanied by an increase in BAT mitochondrial activity, inflammation and oxidative damage.

## Introduction

Obesity and associated comorbidities such as cardiovascular disease, insulin resistance, type 2 diabetes, and nonalcoholic fatty liver disease, among others, are reaching epidemic proportions worldwide^1,2^. However, no effective long-term therapies are currently available. In addition to the relevant contribution of genetic and environmental factors, obesity is ultimately the result of an imbalance between energy intake and energy expenditure. Excess energy is mainly stored in the white adipose tissue (WAT) in the form of triglycerides, but also ectopically in other tissues such as liver, muscle and pancreas, which interferes with their normal function^3^. In recent decades, the adipocyte-centric perspective has gained greater relevance in the mechanisms involved in obesity-related disorders. Adipose tissue is classified into energy-storing WAT and thermogenic-controlling brown adipose tissue (BAT), which burns fatty acids to produce heat and maintain body temperature. Thus, adipose tissue plays a key role in the control of energy homeostasis, through the balance between energy storage and expenditure.

The pathophysiology of obesity-induced metabolic diseases has been attributed to ectopic fat deposition^3^ and WAT dysfunction including increased inflammation^4^, endoplasmic reticulum (ER) and oxidative stress^5,6^, hypoxia^7^, mitochondrial dysfunction^8^, fibrosis and impaired adipocyte expansion and angiogenesis^9–12^. These contributors have been described in WAT, but little is known about the mechanisms involved in BAT during obesity progression. In the last decade, BAT has emerged as a key player in the control of energy metabolism as an active, endocrine organ^13–20^. Brown adipocytes contain multilocular lipid stores, which provide a rapid source of fatty acids that are burned to produce heat and maintain body temperature, *i.e*. thermogenesis. BAT thermogenesis is activated via the β3-adrenergic pathway and takes place in its numerous mitochondria, which contain the BAT-specific uncoupling protein, UCP1. Brown adipocytes are also characterized by high expression of type 2 iodothyronine deiodinase (DIO2), zinc finger of the cerebellum protein (ZIC1), the lipolytic regulator cell death-inducing DNA fragmentation factor α-like effector A (CIDEA), the fibroblast growth factor 21 (FGF21), and the transcription co-regulators PR domain-containing 16 (PRDM16) and peroxisome proliferator-activated receptor gamma coactivator 1α (PGC1α)^21,22^. Several factors have been reported to activate thermogenesis such as natriuretic peptides^23^, bone morphogenetic protein 8b (BMP8b)^24^, norepinephrine^25^, meteorin-like^26^, or FGF21^27^ among others. Importantly, a growing body of evidence identifies BAT as a potential new target against obesity-induced disorders, since it decreases with age and in obese and diabetic patients^15^. A high-fat diet (HFD) has also been shown to alter the molecular networks in BAT in a time-dependent manner^28^. Thus, a study of the mechanisms involved in obesity-induced BAT derangements is needed.

Here we show that BAT from obese mice was hypertrophic and showed higher levels of inflammation, ER stress, reactive oxygen species (ROS) generation and antioxidant enzyme activity than lean counterparts. In addition, an obesity-induced increase in UCP1 protein expression was accompanied by enhanced mitochondrial activity.

## Results

### Phenotypic and histological characterization of HFD-treated mice

**Figure 1:**
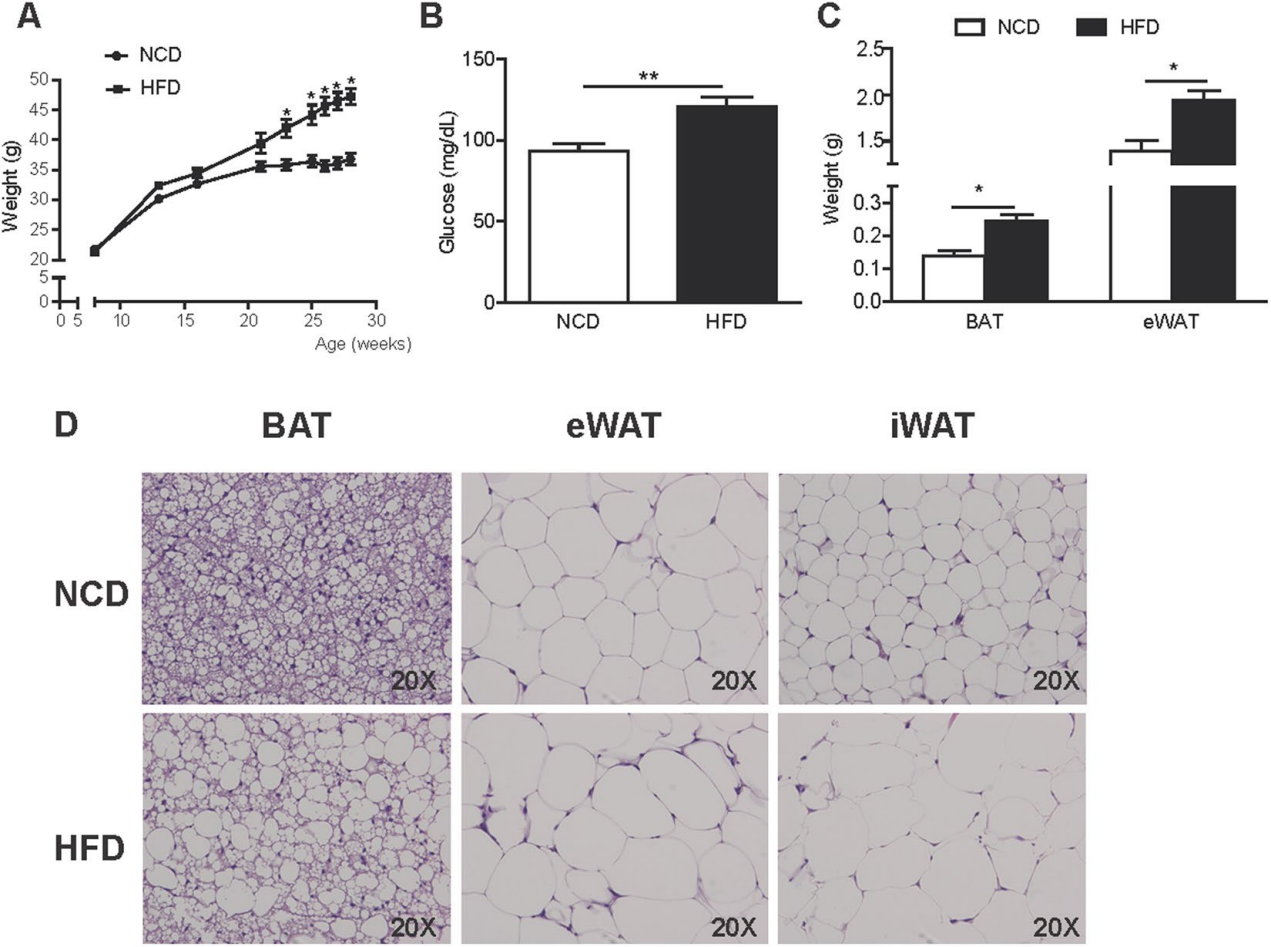
Phenotypic and histological characterization of HFD-treated mice. Changes in body weight (**A**), serum glucose levels (**B**) and adipose tissue weight (**C**) in NCD and HFD-fed mice for 20 weeks. (**D**) Representative histological images from interscapular BAT, eWAT and iWAT from NCD and HFD-fed mice stained with H&E. Results are represented as mean ± SEM. n = 8–10; *p < 0.05; **p < 0.01.

Mice treated with HFD for 20 weeks developed obesity and hyperglycemia (Fig. 1A and B). This time point was used for the following experiments. The weight of interscapular BAT (NCD: 0.14 ± 0.02 g *vs*. HFD: 0.25 ± 0.02 g, *p* = 0.0003) and epididymal WAT (eWAT) (NCD: 1.39 ± 0.12 g *vs*. HFD: 1.93 ± 0.11 g, *p* = 0.003) was higher in obese mice, which indicates hypertrophy of the tissues (Fig. 1C). Histological examination corroborated the hypertrophy of both brown (BAT) and white adipocytes (eWAT and inguinal WAT (iWAT)) under HFD (Fig. 1D). BAT from obese mice showed more unilocular adipocytes compared with the more multilocular adipocytes present in normal chow diet (NCD) mice.

### Increased immune cell infiltration in BAT from obese mice

**Figure 2:**
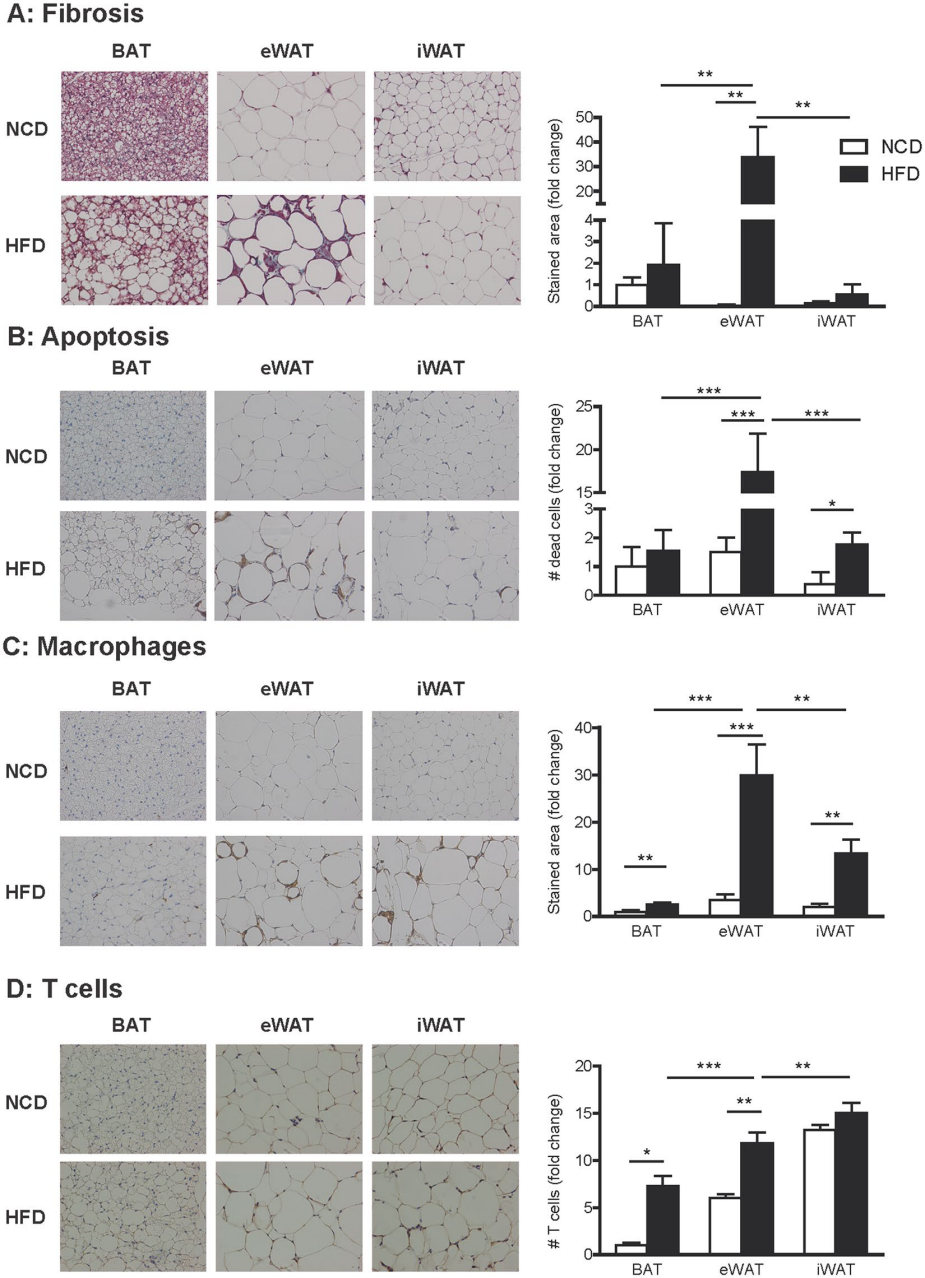
Increased immune cell infiltration in BAT from obese mice. Histological sections from interscapular BAT, eWAT and iWAT were analysed. (**A**) Representative images of Masson´s trichrome staining (left). Total collagen was quantified and expressed as the percentage of stained area in blue (right). (**B**) Representative images of immunohistochemical localization of Caspase-3 (left). The stained area was quantified and apoptosis is expressed as the number of dead cells (right). (**C**) Representative images of immunohistochemical localization of Mac-2 (left). The stained area was quantified and macrophage infiltration was expressed as a percentage of the stained area (right). (**D**) Representative images of immunohistochemical localization of CD3 (left). T cell infiltration was expressed through the quantification of CD3 positive cells (right). Arrows point to stained areas. Results are represented as mean ± SEM. n = 8–10; *p < 0.05; **p < 0.01.

Next, we performed a deeper histological BAT examination looking for signs of fibrosis, apoptosis and inflammation. Despite its hypertrophy, no signs of fibrosis were found in BAT from obese mice, while fibrosis was clearly seen in eWAT from obese mice (Fig. 2A). In fact, eWAT from obese mice showed the highest rate of fibrosis compared to the other obese fat depots. Apoptosis (measured as Caspase 3 staining) was present in both eWAT and iWAT from obese mice (Fig. 2B). However, the degree of apoptosis in BAT from obese mice was lower than in WAT, and was not statistically different from lean controls. The effect of HFD was more pronounced in eWAT, since this tissue exhibited the highest level of apoptosis of the three fat depots studied. The number of infiltrated macrophages and T cells was higher in BAT from obese mice than in lean mice, which indicated the presence of HFD-induced inflammation in BAT (Fig. 2C and D). When comparing the three obese fat depots, BAT showed the lowest number of infiltrated inflammatory cells, while more macrophages where seen in eWAT and T cells in iWAT (Fig. 2C and D).

### Diet-induced obesity increases ER stress and inflammation in BAT

**Figure 3:**
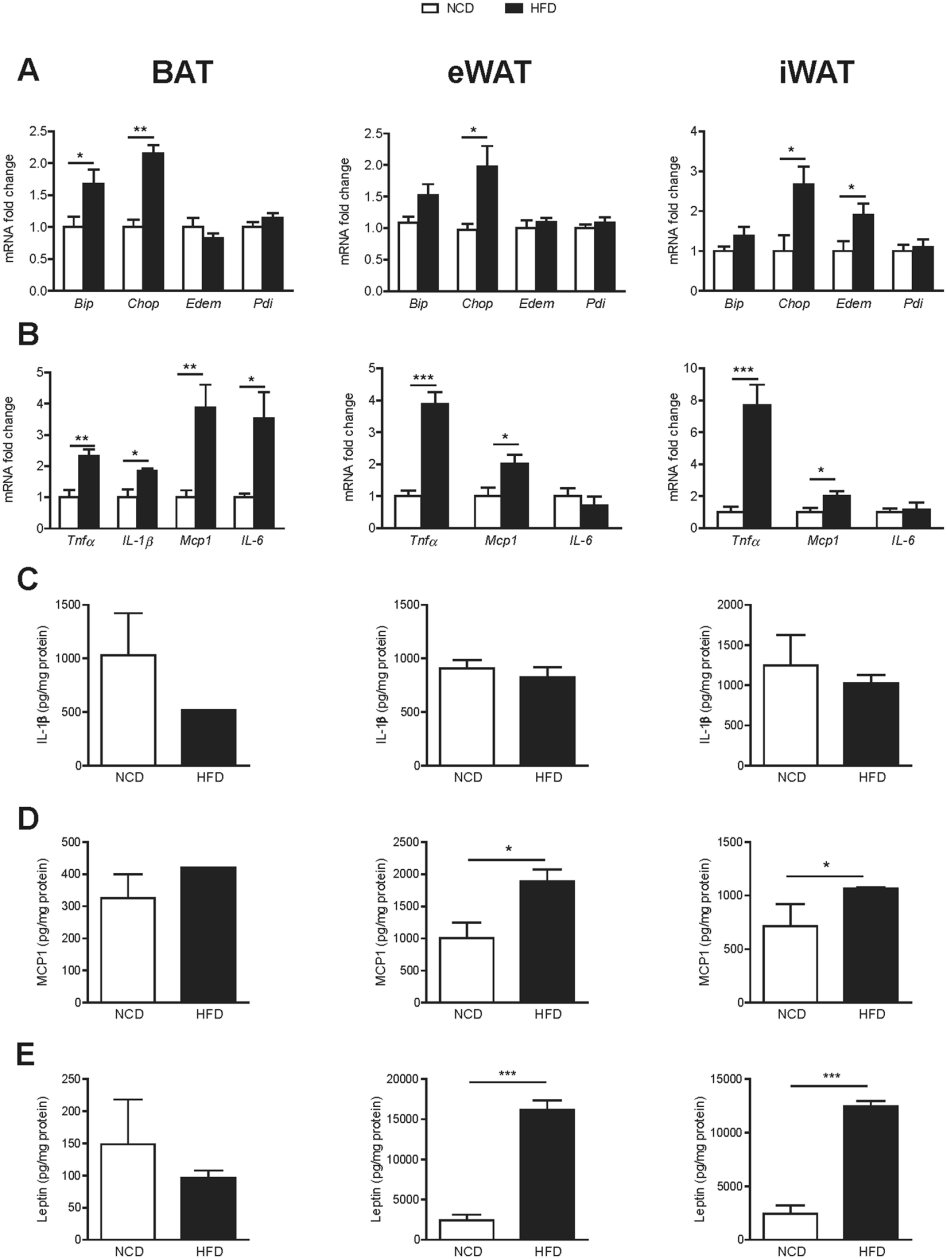
Diet-induced obesity increases ER stress and inflammation in BAT, eWAT and iWAT. (**A**) Endoplasmic reticulum stress, determined as mRNA relative expression of *Bip, Chop, Edem* and *Pdi* by qPCR in interscapular BAT (left), eWAT (middle) and iWAT (right). (**B**) Pro-inflammatory cytokines, determined as mRNA relative expression of *Tnfα, Il-1β, Mcp1* and *Il-6* by qPCR in interscapular BAT (left), eWAT (middle) and iWAT (right). Protein levels of (**C**) IL-1β, (**D**) MCP-1 and (**E**) leptin in interscapular BAT (left), eWAT (middle) and iWAT (right). Results are represented as mean ± SEM. n = 8–10; *p < 0.05; **p < 0.01; ***p < 0.005.

The mRNA expression levels for ER stress (*Bip* and *Chop*) and proinflammatory (*Tnfα*, *Il-1β*, *Mcp1* and *Il-6*) markers were elevated in BAT from HFD animals (Fig. 3A and B). As expected, signs of ER stress and inflammation were found in eWAT and iWAT. However, at protein level, no changes in IL-1β, MCP1 and leptin were found in BAT from HFD mice, while MPC1 and leptin were increased in eWAT and iWAT (Fig. 3C–E).

### BAT from obese mice showed increased ROS generation with a concomitant increase in antioxidant enzyme activity

**Figure 4:**
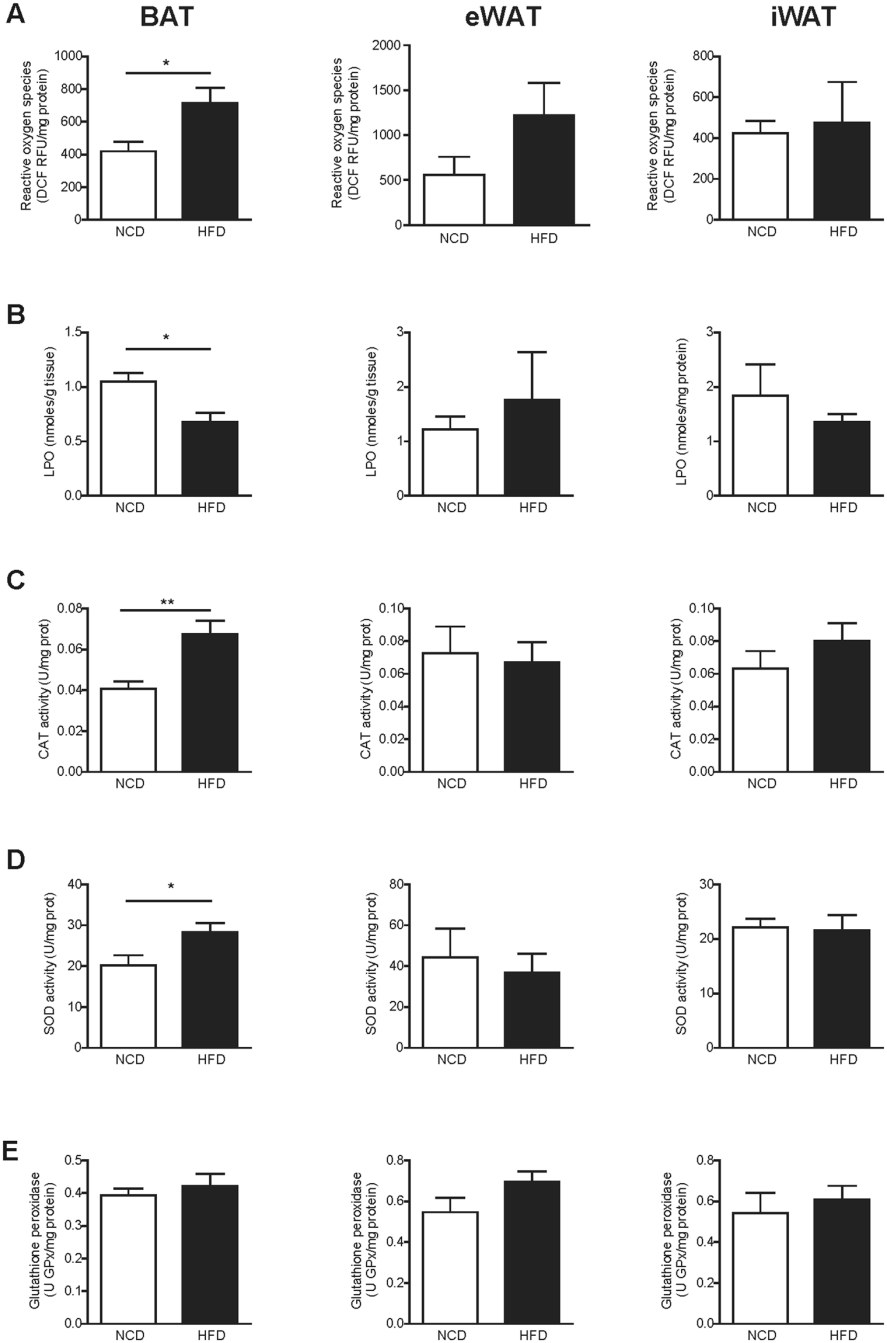
BAT from obese mice showed increased ROS generation with a concomitant increase in antioxidant enzyme activity. Oxidative status in interscapular BAT (left), eWAT (middle) and iWAT (right) from NCD and HFD mice was assessed by determining ROS generation, oxidative damage to lipids, and antioxidant enzymes activity. (**A**) Total ROS were measured by determining the fluorescent probe DCFH-DA oxidation (DCF RFU: Dichlorofluorescein Relative Fluorescence Units). (**B**) Combined detection of malondialdehyde and 4-hydroxynonenal as major lipid peroxidation by-products, expressed together as lipoperoxides (LPO). (**C**–**E**) Antioxidant activity of the enzymes catalase (CAT) (**C**), superoxide dismutase (SOD) (**D**) and glutathione peroxidase (**E**). Results are represented as mean ± SEM. n = 8–10; *p < 0.05; **p < 0.01.

Next, we assessed oxidative status in BAT, eWAT and iWAT from NCD and HFD mice by determining ROS generation, oxidative damage to lipids (lipoperoxides; LPO) and the activity of the antioxidant enzymes catalase (CAT), superoxide dismutase (SOD) and glutathione peroxidase (Fig. 4). BAT from obese mice showed higher levels of ROS generation (Fig. 4A, NCD: 419.30 ± 59.73 Dichlorofluorescein Relative Fluorescence Units (DCF RFU)/mg protein *vs*. HFD: 712.60 ± 95.09 DCF RFU/mg protein, *p* = 0.03) and antioxidant enzyme activity (Fig. 4C and D) than lean counterparts. No changes were seen in glutathione peroxidase activity (Fig. 4E). LPO levels were decreased in BAT under HFD (Fig. 4B). No differences were seen in eWAT and iWAT.

### HFD increases UCP1 protein levels without changes in BAT mitochondrial content

**Figure 5:**
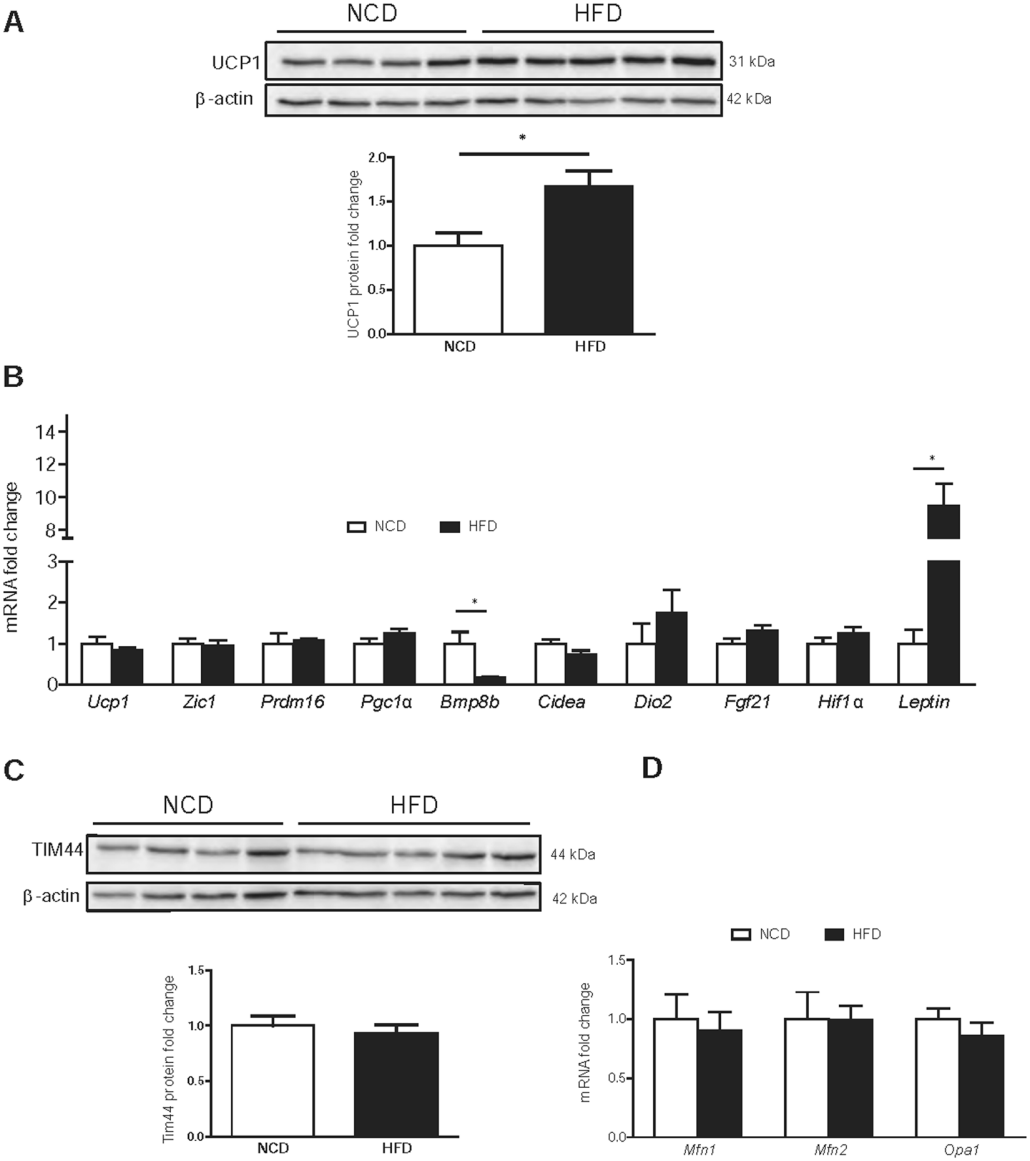
HFD increases UCP1 protein levels without changes in BAT mitochondrial content. Whole interscapular BAT lysates were subjected to Western Blot analysis. (**A**) UCP1 protein levels using β-actin as a loading control. (**B**) Relative mRNA expression of BAT markers *Ucp1, Zic1, Prdm16, PGc1α, Bmp8b, Cidea, Dio2, Fgf21, Hif1α* and *Leptin* in NCD and HFD-fed mice. (**C**) TIM44 protein levels using β-actin as a loading control. Shown representative immunoblots out of at least 4 independent experiments. (**D**) Mitochondrial dynamics were determined by the relative mRNA expression of *Mfn1, Mfn2* and *Opa1* of BAT in NCD and HFD-fed mice. Results are represented as mean ± SEM. n = 8–10; *p < 0.05.

Protein levels of the BAT-specific marker UCP1 were increased under HFD (Fig. 5A, UCP1 protein fold change: 1.00 ± 0.14 A.U. *vs*. 1.67 ± 0.18 A.U., *p* = 0.027 for NCD and HFD, respectively). However, no changes were seen at mRNA level (Fig. 5B). The mRNA expression of *Bmp8b* was reduced in BAT from obese mice, and *Leptin* levels were elevated (Fig. 5B). No changes were seen in other BAT markers such as *Zic1*, *Prdm16*, *Pgc1α, Cidea, Dio2, Fgf21* and *Hif1α*. To evaluate whether HFD led to an increase in mitochondrial content, we measured the expression of TIM 44 (translocase of mitochondrial inner membrane 44, a mitochondrial content marker), and mitofusin (*Mfn*) 1, *Mfn2* and *Opa1* (proteins implicated in mitochondrial dynamics and biogenesis, and target genes of PGC1α). No changes were seen in any of these markers, which indicates that, in our conditions, HFD did not affect mitochondrial content or biogenesis (Fig. 5C and D).

### Enhanced mitochondrial respiration in BAT from obese mice

**Figure 6:**
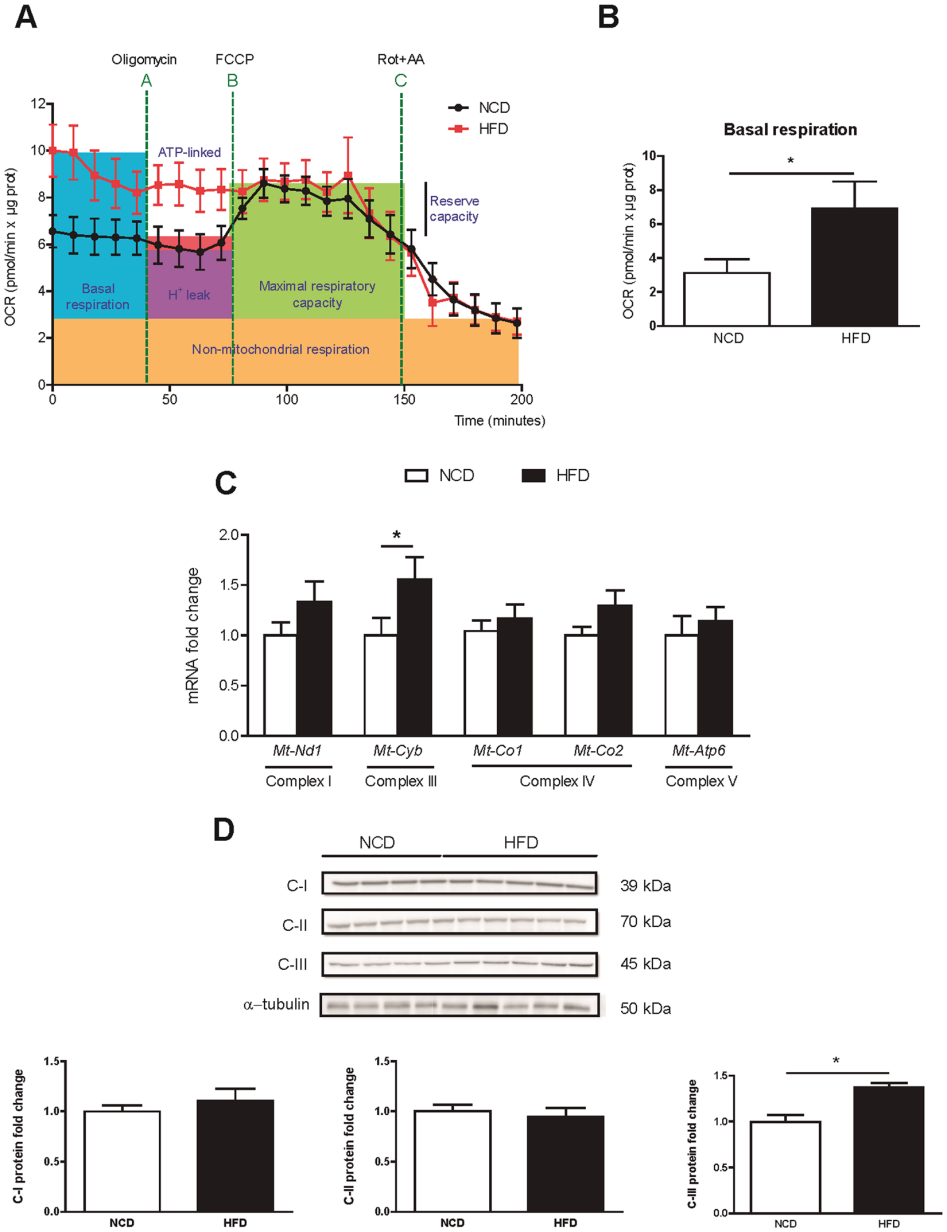
Enhanced mitochondrial respiration activity in BAT from obese mice. (**A**) Bioenergetic profile of interscapular BAT explants in NCD (black dots) and HFD-fed (red squares) mice. Nine milligrams of BAT/mice were analysed using the XF24 islet capture microplate. Basal oxygen consumption is recorded. After oligomycin was added in A, ATP-linked respiration and H^+^ leak were calculated. Maximal respiration and reserve capacity were measured after FCCP addition, and finally ETC inhibitors were added in C to determine non-respiratory oxygen consumption. Concentration of inhibitors and incubation times are described in the materials and methods section. Results are normalized by protein content. (**B**) Quantification of basal respiration in NCD and HFD mice. (**C**) Relative mRNA expression of the mitochondrial ETC complexes I, III, IV and ATP synthase. (**D**) Whole BAT lysates were subjected to Western blot. Complexes I, II and III protein levels were analysed by immunoblot, using α-tubulin as a loading control. Results are represented as mean ± SEM. n = 8–10; *p < 0.05.

Mitochondrial bioenergetics were directly measured in BAT explants with XF Seahorse technology, as previously described^29^. Lean BAT showed a typical bioenergetic profile (Fig. 6A). The oxygen consumption rate (OCR) decreased after the injection of oligomycin (an ATP synthase inhibitor), which reveals the amount of O_2_ consumed by H + leak, ATP synthesis and other oxidases. The addition of the uncoupling agent carbonylcyanide p-triflouromethoxyphenylhydrazone (FCCP) forced the maximal respiration capacity of the BAT. Finally, a mix of rotenone and antimycin A, which are inhibitors of complexes I and III of the mitochondrial electron transport chain (ETC), fully depleted mitochondrial O_2_ consumption. In contrast, BAT from obese mice presented its maximal respiratory capacity from the beginning of the experiment. The basal respiratory rate was 2-fold higher in BAT from obese mice than in the lean controls (Fig. 6B, OCR (pmol/min × μg prot): 3.12 ± 0.81 *vs*. 6.92 ± 1.58, *p* = 0.03 for NCD and HFD, respectively). In addition to this maximal respiration in basal conditions, BAT from obese mice was unresponsive to the addition of oligomycin and FCCP, and only inhibition of the ETC respiratory complexes I and III managed to reduce O_2_ consumption. In fact, both the mRNA levels (Fig. 6C) and the protein levels (Fig. 6D) of the mitochondrial ETC complex III (cytochrome b) were elevated in BAT from obese mice (Fig. 6C). No changes were seen in the other ETC complexes, such as mitochondrial NADH dehydrogenase 1 (Mt-Nd1, complex I), cytochrome C oxidase I (Mt-Co1, complex IV component), and ATP synthase 6 (Mt-ATP6, complex V) (Fig. 6C and D).

## Discussion

Obesity-associated WAT fibrosis, inflammation, oxidative damage, apoptosis and mitochondrial dysfunction contributes to the dysregulation of WAT metabolism and the development of metabolic diseases such as insulin resistance and type 2 diabetes. However, whether these mechanisms occur in BAT with obesity and alter its function is unknown.

Here we treated mice with HFD until they reached obesity and hyperglycemia. At that time point, BAT from obese mice was hypertrophic, *i.e*. had increased tissue weight and enlarged adipocytes. A histological analysis of BAT revealed that the lipid accumulation was not enough to induce fibrosis or apoptosis, as it did in eWAT and iWAT. Consistent with this, mRNA expression of the *Hif1α* did not increase in BAT from obese mice. However, if obesogenic conditions were extended over time, it is possible that extracellular matrix accumulation would increase, and BAT mitochondria could begin to present signs of malfunction. McGregor and col. showed an increase in fibrosis-related gene expression after 20–24 weeks of HFD^28^. This may suggest that the expandability hypothesis that has been described for WAT may also apply to BAT^30^.

The infiltration of immune cells such as macrophages and T cells increased in BAT from obese mice, although to a lesser extent than in eWAT and iWAT. qPCR analysis also revealed an increase in proinflammatory cytokine gene expression in BAT, eWAT and iWAT, although no differences were yet evident at protein level in BAT. This suggests that the inflammatory response in this tissue is slower than in WAT. The inflammatory environment indicated that unresolved inflammation is also present in BAT from obese mice. Whether BAT in C57BL6 obese mice is resistant to inflammation appears to be a time-related issue. While short-term diet-induced obesity did not promote cytokine release or immune cell migration^31^, the upregulation of proinflammatory genes seems to be triggered after 24 weeks of HFD, which coincides with the upregulation of fibrosis markers^28^. Genetic models of obesity have also revealed the presence of inflammation in BAT^32^.

ER stress is also a typical feature of obesity. It has been described as a mechanism that attenuates adaptive thermogenesis in BAT^33,34^. According to our results, ER stress was enhanced in BAT of HFD-treated mice, as shown by the increase in the mRNA expression of *Bip* and *Chop*. In our study, obesity-related pathological processes such as apoptosis, fibrosis, inflammation or ER stress are more evident in obese WAT than in BAT. The exception was ROS generation, which has also been widely related to obesity^6,35^. While no changes were found in ROS generation or antioxidant defence in both WAT depots, BAT from obese mice showed a nearly 2-fold increase in ROS generation due to higher metabolic activity. As a countermeasure, both CAT and SOD activities were increased, which successfully prevented oxidative damage to macromolecules. In fact, the levels of oxidative damage to LPO were lower than those found in the lean animals. This effect has been described^36^ previously, and it is probably caused by increased content of antioxidant vitamin E in the HFD diet (NCD: 49.3 IU/Kg *vs*. HFD: 67.2 IU/Kg), which could confer some protection against lipid peroxidation. Altogether, this points to BAT as an active contributor to obesity-induced production of oxidative damage.

The main finding from our study is that the mitochondrial respiration of BAT from obese mice, at the measured time point, is working at its maximal capacity, consuming more than twice the amount of O_2_ as lean counterparts. BAT activation in obesity has been a matter of debate for the past few years. First, there is no unified method to assess BAT activity. Indirect methods have been used, such as recording the electrical activity in nerves innervating BAT^37^, measuring epinephrine turnover in the synapsis^38^, mitochondrial size^39^ or adrenergically activated gene expression analysis^40^. Direct methods, such as recording oxygen consumption, require mitochondria or primary adipocyte isolation. To our knowledge, this is the first report that measures bioenergetics directly in BAT explants from obese mice. Working with the whole tissue and not just with subcellular populations or isolated mitochondria, which are prone to rupture, better reflects the physiological state of mitochondria without losing the tissue context. Second, whether activation or depletion of BAT thermogenic activity is produced in obesity is still unclear. In murine models of genetic obesity, a decrease in BAT activation has been reported, probably as a consequence of a concomitant reduction in UCP1^32^. As recently reported^41^, in leptin-deficient mice, leptin acts centrally as an anapyrexic to elevate the threshold temperature for processes such as conductance (heat loss), but does not activate thermogenesis. In diet-induced obese models, where UCP1 levels are kept stable or even increased as we have shown in the present report, BAT activation occurs to trigger the diet-induced adaptive thermogenesis response^42,43^. However, it is yet to be confirmed whether this is a time-sensitive effect. In addition, a number of strategies to increase BAT activity, such as cold, caloric restriction or beta-adrenergic inducers have successfully activated BAT, protecting from obesity and insulin resistance^19,23,25,44^.

In our model, the increased oxidation of energetic substrates induced by HFD enhances the ETC, as inferred by the increased expression of complex III and increased oxygen consumption. Only the addition of inhibitors of this complex managed to decrease oxygen consumption in BAT from obese mice, which highlights the importance of this step in the overall activity of mitochondrial respiration. The accumulated protons in the intermembrane space are pumped back to the mitochondrial matrix via UCP1, which is not only increased in terms of protein content, as described in other models of diet-induced obesity^42^, but may also be activated by FFA^45^. Importantly, enhanced respiration is independent of the number of mitochondria, since we did not find any differences in mitochondrial biogenesis markers such as TIM44, mitofusins (Mfn1 and Mfn2) or PGC1α expression. In fact, no changes were seen under HFD in the mRNA expression of the main BAT markers (*Zic1, Prdm16, Pgc1α, Cidea, Dio2* and *Fgf21*). Surprisingly, the thermogenic activator Bmp8b was decreased in BAT from obese mice, which may suggest altered thermogenic function^46^. However, it has recently been reported that another member of the Bmp protein family, BMP7, requires a cold temperature to activate BAT thermogenesis^46^. Thus, the effect of reduced Bmp8b expression over thermogenesis may be limited in our model of obese mice housed at room temperature. Taken together, these results may suggest that in our model, BAT from obese mice maintains its functionality, working at its maximal capacity as a homeostatic mechanism, to increase fuel oxidation.

In summary, we have shown that the BAT of HFD-treated mice is hypertrophic with higher levels of inflammation, ER stress, ROS generation and antioxidant enzyme activity. Our study also demonstrates how diet-induced adaptive thermogenesis in BAT is caused by maximal activation of mitochondrial respiration and the upregulation of UCP1. Although BAT from obese mice showed an initial state of tissue dysregulation characterized by increased inflammation, ER stress and ROS generation, it was not enough to disrupt its fuel-burning capacity. However, if the obesogenic conditions were maintained, it is possible that the thermogenic capacity of the tissue could be compromised, thus exacerbating obesity and its metabolic consequences.

## Methods

### Animals

Six-week-old male C75Bl/6 J mice were purchased from Janvier Laboratories (France). Mice were maintained for 2 weeks in our facility to acclimatize prior to the experiments. Eight-week-old mice were fed *ad libitum* for 20 weeks with either NCD (TestDiet 58Y2, 10% Kcal fat) or HFD (TestDiet 58Y1, 60% Kcal fat). Two independent sets of animals with n = 8–10 mice were used in each experiment. All mice had free access to water and chow and were housed under alternating 12 h light and dark cycles. Blood samples were obtained from the tails of unanesthetized mice after an overnight fast. Animals were sacrificed by cervical dislocation. Blood glucose concentrations were measured using a Glucometer Elite (Bayer). All experiments were performed in accordance with the European Community Council directive 86/609/EEC and were approved by the Institutional Animal Care and Use Committee of the University of Barcelona.

### Histological analysis

Interscapular BAT, eWAT and iWAT were excised and fixed overnight in 10% PBS-buffered formalin, and were thereafter stored in 50% ethanol. Histological samples were paraffin-embedded and 4 μm thick sections were prepared from each sample, which were stained with hematoxylin and eosin (H&E) at the Pathology Department of the Hospital Clinic of Barcelona. Fibrosis was evaluated with Masson’s Trichrome staining performed at the Tumor Bank of the HCB-IDIBAPS Biobank (IDIBAPS, Barcelona). Two-four μm serial sections were used for immunohistochemistry at the Tumor Bank of the HCB-IDIBAPS Biobank (IDIBAPS, Barcelona), using the Leica Microsystems’ Bond-max™ automated immunostainer, together with the Bond Polymer Refine Detection System (Leica Microsystems, Spain). Briefly, tissue sections were deparaffinized, and pretreated for antigen retrieval with 1 mM EDTA, pH 8.0 for 15 minutes for CD3 or with citrate buffer, pH 6.0 for 20 minutes for Mac2, FOXP3 and Caspase3. For macrophage immunostaining, we used primary monoclonal rat anti-murine antibody to Mac2 (1/40,000; Cedarlane) combined with a secondary rabbit anti-rat Ig (1/3,000). For T cells staining, we used primary polyclonal rabbit anti-murine CD3 antibody (1/4,000; Cell Marque). For apoptosis, we used monoclonal rabbit anti-murine Caspase3 antibody (1/500; Cell Signaling). Finally, samples were developed with diaminobenzidine and counterstained with hematoxylin. Sections were viewed under a U-25ND6 Olympus microscope at × 20 magnification. Photomicrographs were generated with Cell^B^ software and a minimum of 10 high power field/mouse were quantified using Image J analysis software.

### Analysis of mRNA expression by quantitative real-time PCR

Total RNA was isolated from tissues (interscapular BAT, eWAT or iWAT) using RNeasy Lipid Tissue Mini Kit (QIAGEN), and cDNA was obtained using a Transcriptor First Strand cDNA Synthesis kit (Roche), following the manufacturer’s instructions. Relative quantification of mRNA was performed using a LightCycler® 480 instrument (Roche) in 10 μL of reaction medium by using 6.5 ng of cDNA, forward and reverse primers at 100 nM each, and a SYBR Green PCR Master Mix Reagent kit (Life Technologies). Primer pairs are shown in Table 1. The mRNA levels were normalized to those of TBP and expressed as fold change.

**Table 1:** Quantitative real-time PCR oligonucleotides.

	Forward	Reverse
*Bip*	5′-ACTTGGGGACCACCTATTCCT-3′	5′-ATCGCCAATCAGACGCTCC-3′
*Bmp8b*	5′-ATGCGAGTCCGCTAAACG-3′	5′-GGCCCAGTAGCCATAGGAGT-3′
*Chop*	5′-CCCTGCCTTTCACCTTGG-3′	5′-CCGCTCGTTCTCCTGCTC-3′
*Cidea*	5′-GCCTGCAGGAACTTATCAGC-3′	5′-AGAACTCCTCTGTGTCCACCA-3′
*Dio2*	5′-CCTTGGTCCCCCACTTCT-3′	5′-GCTTCCCCAGTCACCTTCTT-3′
*Edem*	5′-AAGCCCTCTGGAACTTGCG -3′	5′-AACCCAATGGCCTGTCTGG-3′
*Fgf21*	5′-GGGCTTCAGACTGGTACACAT-3′	5′-AGATGGAGCTCTCTATGGATCG-3′
*Hif-1α*	5′-CAGGATCAATGACATTTCACACA-3′	5′-GCTGGTGAGGACCTGTTGAT-3′
*Ifn-γ*	5′-ATCTGGAGGAACTGGCAAAA-3′	5′-TTCAAGACTTCAAAGAGTCTGAGGTA-3′
*Il-1α*	5′-TTGGTTAAATGACCTGCAACA-3′	5′-GAGCGCTCACGAACAGTTG-3′
*Il-1β*	5′- GCCCATCCTCTGTGACTCAT-3′	5′-AGGCCACAGGTATTTTGTCG-3′
*Leptin*	5′-GCACTAGACAAAGTTCACCTGAGA-3′	5′-CACCTGGGCAGCATAGGA-3′
*Mcp1*	5′-ATGGCACTTCTCTTGCCTTC-3′	5′-GTCGGGAGTGTGGTTCAGAC-3′
*Mfn1*	5′-GTGAGCTTCACCAGTGCAAA-3′	5′-CACAGTCGAGCAAAAGTAGTGG-3′
*Mfn2*	5′-CATTCTTGTGGTCGGAGGAG-3′	5′-AAGGAGAGGGCGATGAGTCT-3′
*Mt-atp6*	5′-CAGTCCCCTCCCTAGGACTT-3′	5′-TCAGAGCATTGGCCATAGAA-3′
*Mt-co1*	5′-ACTATACTACTAACAGACCG-3′	5′-GGTTCTTTTTTTCCGGAGTA-3′
*Mt-co2*	5′-ACCTGGTGAACTACGACTGCTAGA-3′	5′-TGCTTGATTTAGTCGGCCTGGGAT-3′
*Mt-cytb*	5′-ACCAATCTCCCAAACCATCA-3′	5′-TCCAGAGACTTGGGGATCTAAC-3′
*Mt-nd1*	5′-GTTGGTCCATACGGCATTTT-3′	5′-TGGGTGTGGTATTGGTAGGG-3′
*Opa1*	5′- TTCTGAGGCCCTTCTCTTGT-3′	5′-TGATCTGTTGCTCGAAATGC-3′
*Pdi*	5′-ACCTGCTGGTGGAGTTCTATG-3′	5′-CGGCAGCTTTGGCATACT-3′
*Pgc1α*	5′-GAAAGGGCCAAACAGAGAGA-3′	5′-GTAAATCACACGGCGCTCTT-3′
*Prdm16*	5′-CCTAAGGTGTGCCCAGCA-3′	5′-CACCTTCCGCTTTTCTACCC-3′
*Tbp*	5′-ACCCTTCACCAATGACTCCTATG-3′	5′-TGACTGCAGCAAATCGCTTGG-3′
*Tnfα*	5′-TTTGAGATCCATGCCGTTG-3′	5′-CTGTAGCCCACGTCGTAGC-3′
*Ucp1*	5′-AGGCTTCCAGTACCATTAGGT-3′	5′-CTGAGTGAGGCAAAGCTGATTT-3′
*Zic1*	5′-AACCTCAAGATCCACAAAAGGA-3′	5′-CCTCGAACTCGCACTTGAA-3′

### Oxidative damage determination

Tissues homogenates in PBS with 5 mM BHT were used to determine oxidation to lipids and proteins. Lipid peroxidation was determined using Bioxytech LPO-586kit (OxisResearch). Advanced oxidation protein products were determined according to Witko-Sarsat’s method, with minor modifications^47^.

### Antioxidant enzymes

Catalase (CAT) specific activity was measured by monitoring the disappearance of hydrogen peroxide at 240 nm. To measure superoxide dismutase (SOD) enzymatic activity, we adapted the method described by Bamforth^48^. It is based on the xanthine/xanthine oxidase reaction for superoxide anion generation (O_2_−) and reduction of cytochrome c for detection at 546 nm. The glutathione peroxidase (GPx) specific activity assay is based on the oxidation of glutathione by GPx. Oxidized glutathione is regenerated by glutathione reductase, using NADPH + H^+^ as a cofactor. The reaction rate was measured following the disappearance of NADPH + H^+^ at 340 nm.

### Western blot analysis

Frozen tissue was homogenized in protein extraction buffer (30 mM Hepes, pH 7.4, 150 mM NaCl, 10% glycerol, 1% Triton X-100, 0.5% sodium deoxycholate [DOC] with phosphatase inhibitors and protease inhibitors). Protein concentration was determined using a BCA protein assay kit (Thermoscientific). Samples were separated on 8% and 12% SDS-PAGE gels and then transferred onto PVDF membranes (Millipore). The following primary antibodies were used: TIM44 (1/5,000; BD Bioscience), UCP1 (1/1,000; Abeam), mitochondrial complexes I, II, III, IV and V (1/5,000; Life Technologies), α-tubulin (1/5,000; Abcam) and β-actin (1/50,000; Sigma-Aldrich). Blots were incubated with the appropriate IgG-HRP-conjugated secondary antibody. Protein bands were visualized using the ECL immunoblotting detection system (GE Healthcare) and developed on an ImageQuant LAS4000 mini Fuji luminescence imagining system. For the analysis of protein expression, bands from at least three independent experiments were quantified by densitometry using Image J analysis software.

### Cytokines measurement

Tissue levels of IL-6, TNF-α, IL-1β and MCP-1 were measured using a Mouse Luminex Screening Assay (Mouse premixed multianalyte kit, R&D Diagnostics, Minneapolis, USA) with polystyrene beads and analyzed with a Luminex100 system and the accompanying Bio-Plex ManagerTM Software 6.1(Bio-Rad, Hercules, California, USA) according to manufacturer’s instructions.

### Seahorse bioanalyzer

The Seahorse XF24 (Seahorse Bioscience, www.seahorsebio.com) was used to measure oxygen consumption rate (OCR) in a whole interscapular BAT explant, as previously described^29^. Briefly, for a bioenergetic profile, we used oligomycin to block ATP synthase; the uncoupler carbonyl cyanide-4-(trifluoromethoxy)phenylhydrazone (FCCP) to measure maximal respiratory capacity; followed by rotenone, the complex 1 inhibitor, and antimycin-A to leave only non-mitochondrial activity to be measured (all from Sigma-Aldrich). Before the measurement, the microplate containing the tissues in DMEM plus 5 mM glucose was incubated at 37 °C without CO_2_ for 45 minutes. During the assay, we injected the following at the final concentrations shown: 24 µg/ml oligomycin, 0.8 μM FCCP, 5 μM rotenone and 15 μM antimycin-A. OCR was calculated by plotting the O_2_ tension of media as a function of time (pmol/min), and data were normalized by the protein concentration measured in each individual well.

### Statistical analysis

Data are expressed as the mean ± standard error of mean (SEM). Statistical significance was defined as p ≤ 0.05 using the following tests: unpaired Student’s *t*-test to analyse differences between the NCD and HFD groups and two-way ANOVA with Bonferroni post hoc analysis for differences in immunohistological staining between NCD and HFD groups and among the different tissues. All figures and statistical analyses were generated using GraphPad Prism 6 software.

## Electronic supplementary material

Supplemental information
