# Supplementary material for: Increased inflammation, oxidative stress and mitochondrial respiration in brown adipose tissue from obese mice
Source: Sci Rep. 2017 Nov 22;7:16082. doi: 10.1038/s41598-017-16463-6 (PMC5700117; doi:10.1038/s41598-017-16463-6)
Supplement: Supplementary file 1 — Supplemental information [file 41598_2017_16463_MOESM1_ESM.pdf]

SUPPLEMENTAL INFORMATION

Increased inflammation, oxidative stress and mitochondrial respiration in brown adipose tissue from obese mice

Martín Alcalá[1,#], María Calderon-Dominguez[2,3,#,&], Eduviges Bustos[2,$], Pilar Ramos[1], Núria Casals[4], Dolors Serra[2,3*], Marta Viana[1*], Laura Herrero[2,3*]

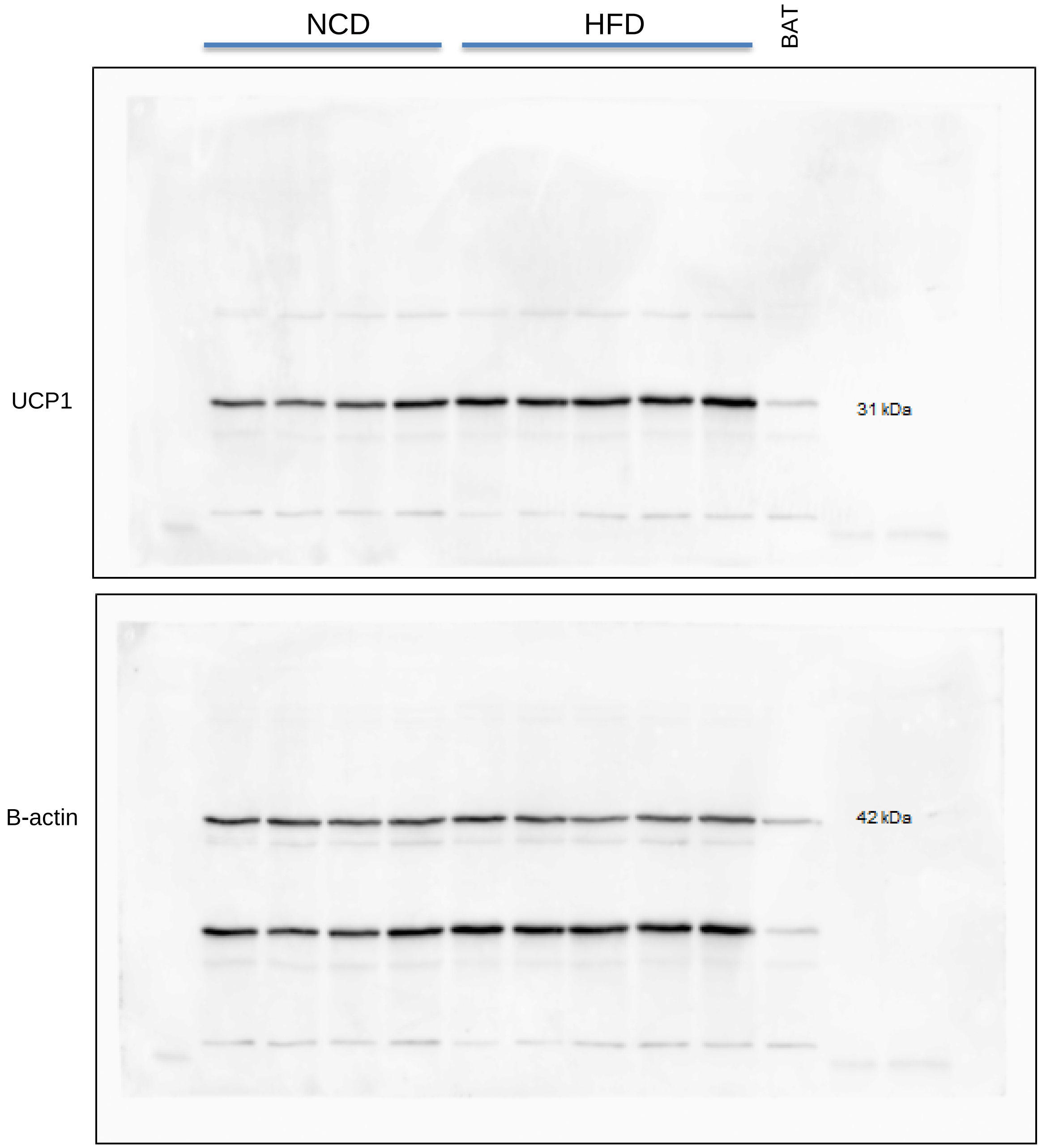
NCD
HFD
BAT
UCP1
31 kDa
B-actin
42 kDa

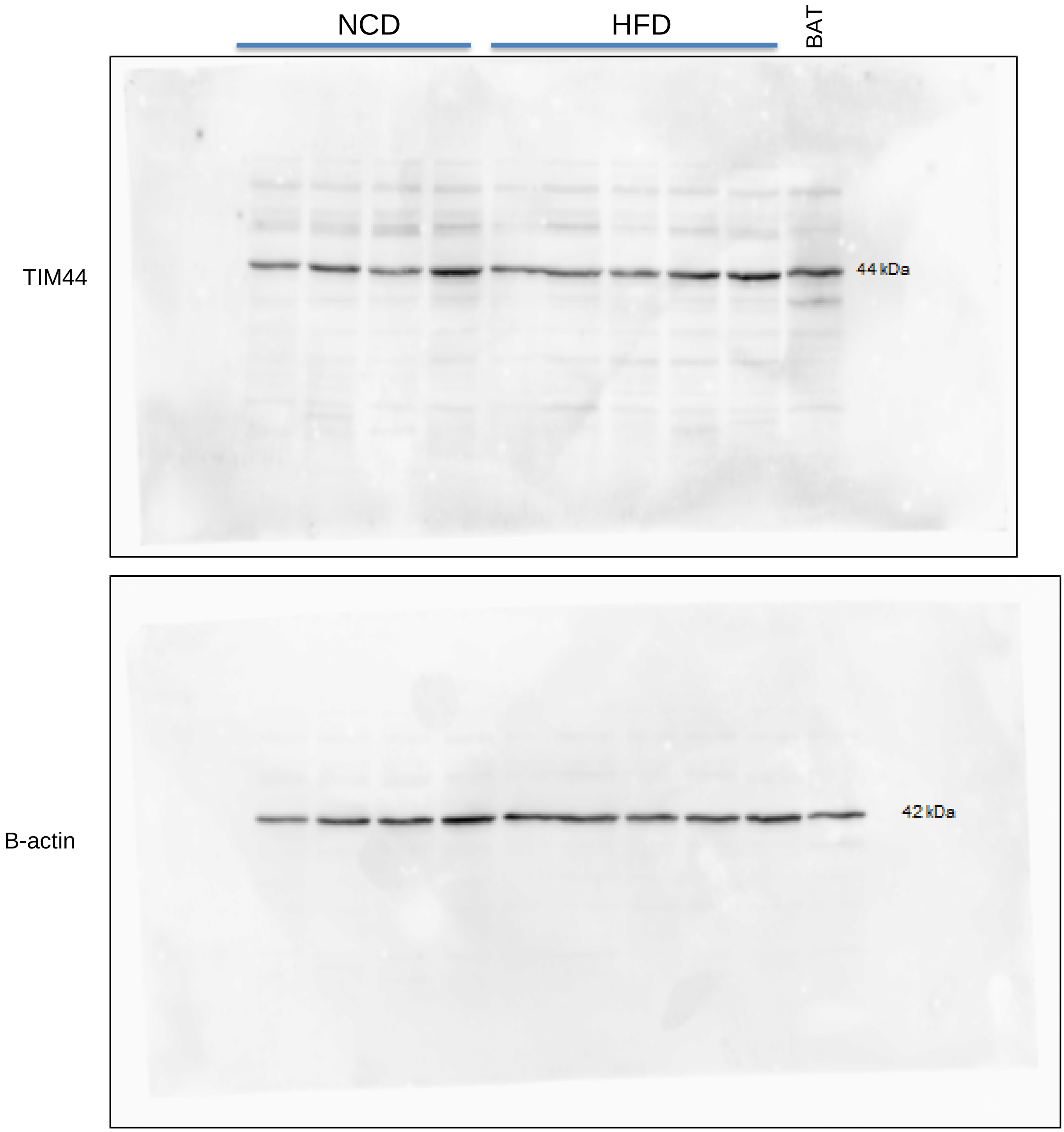
NCD
HFD
BAT
TIM44
44 kDa
B-actin
42 kDa

Mitochondrial ETC complexes I, II, III

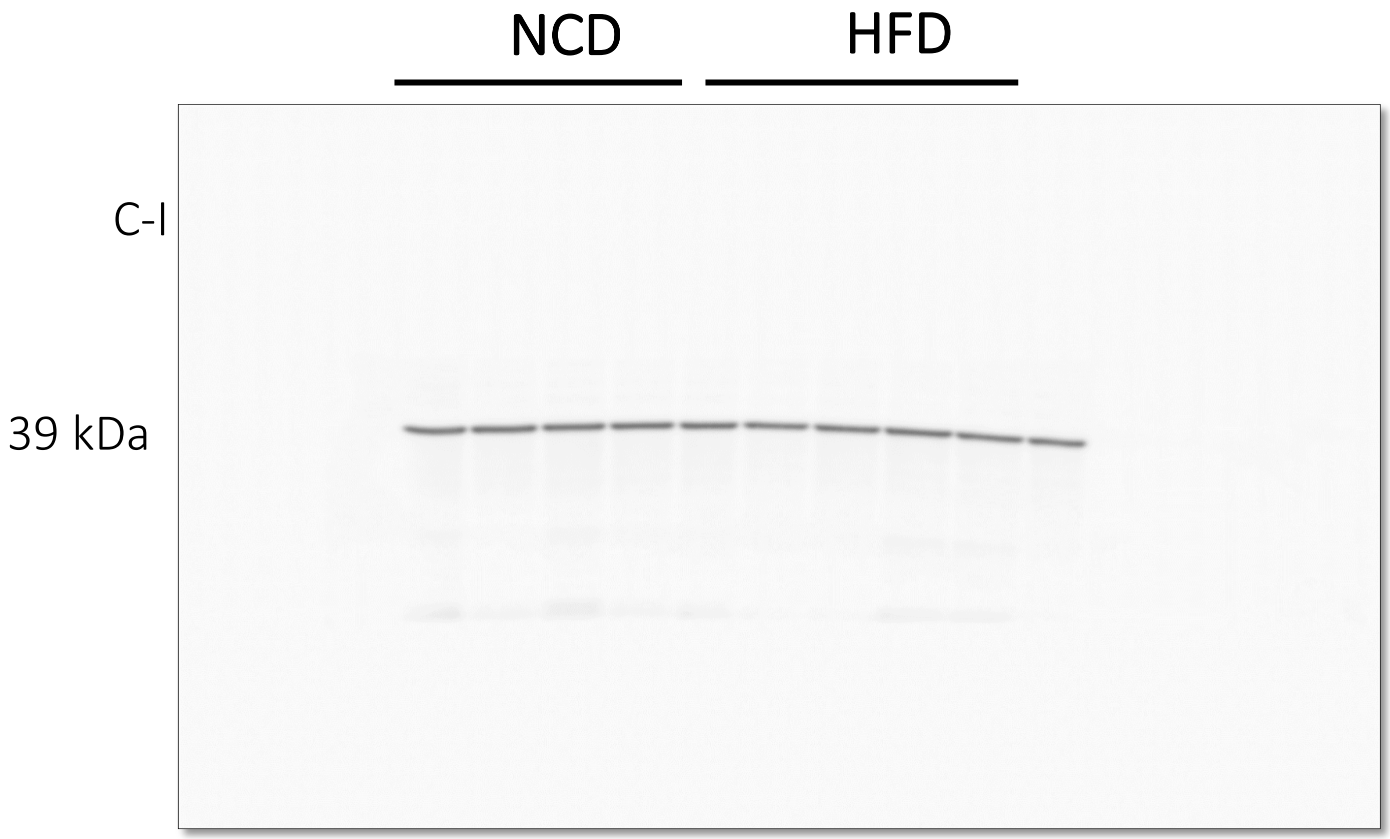

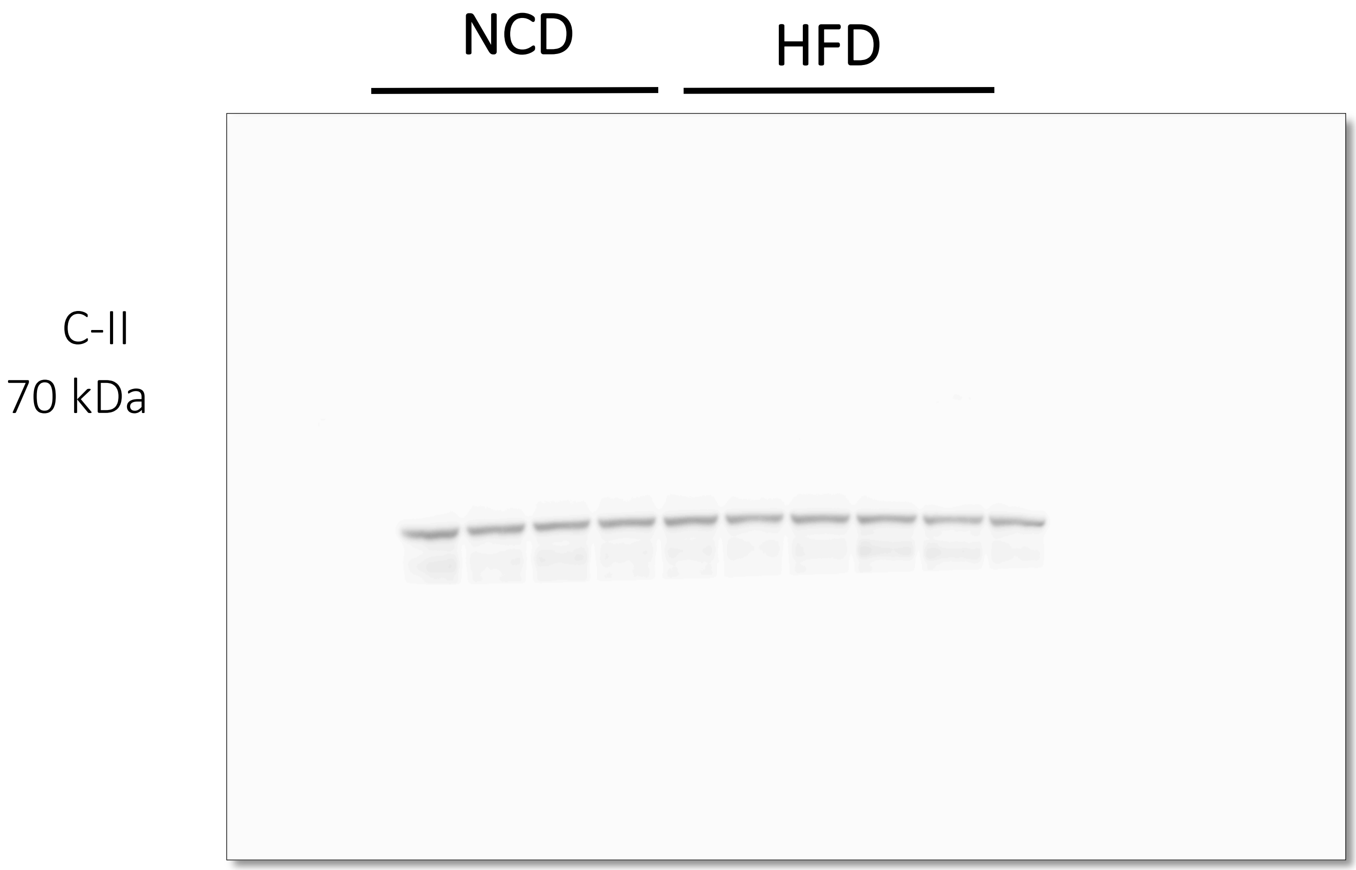
NCD
HFD
C-II
70 kDa

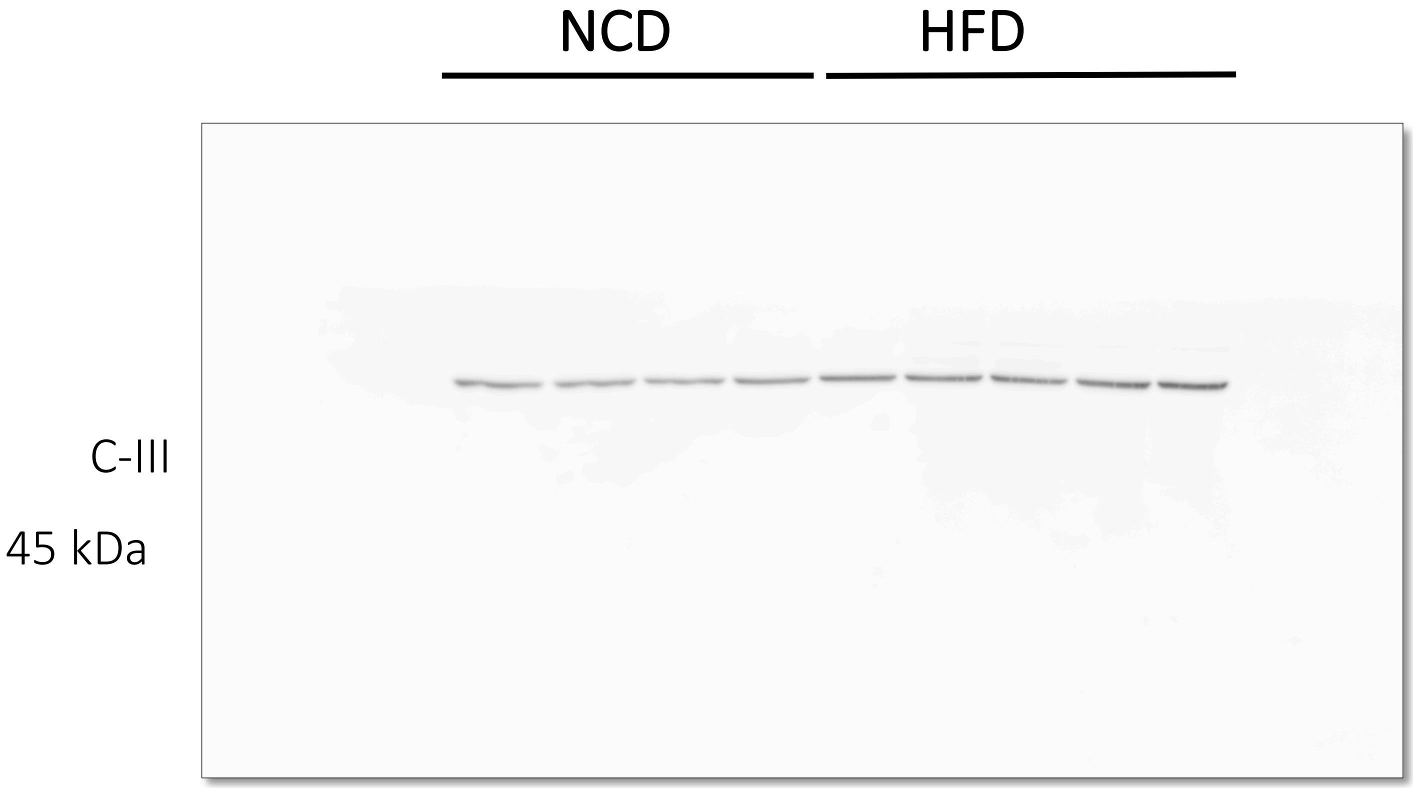
NCD
HFD
C-III
45 kDa

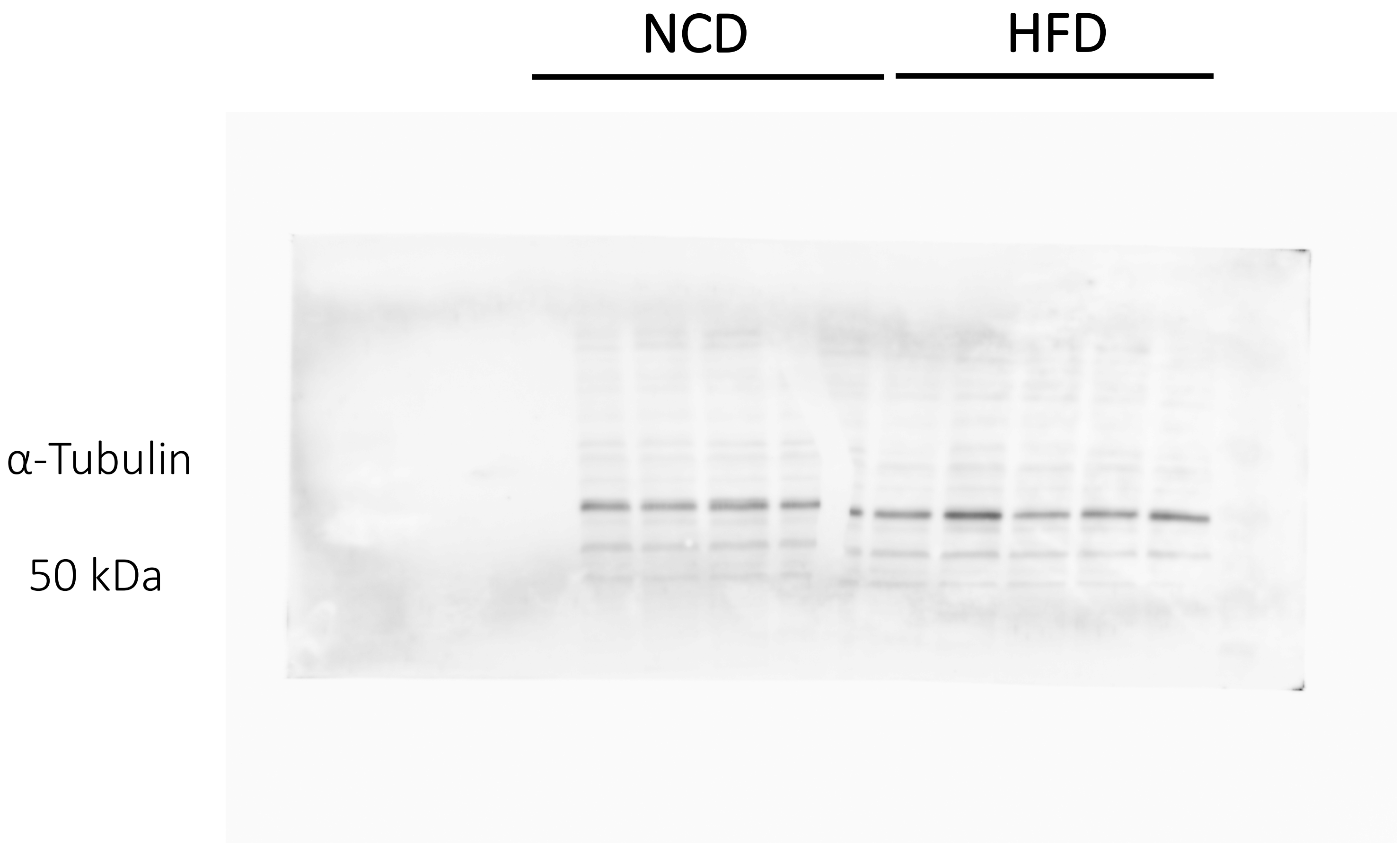
NCD
HFD
α-Tubulin
50 kDa
